# Comparative evaluation of reversed-phase and hydrophilic interaction liquid chromatography columns for untargeted profiling of bioactive compounds in* Hypericum perforatum*

**DOI:** 10.1007/s00216-025-06030-8

**Published:** 2025-07-26

**Authors:** Davide Barboni, Desiree Bozza, Damiana Natasha Spadafora, Nicoletta Bianchi, Brunilda Myftari, Paola Tedeschi, Chiara De Luca, Simona Felletti, Matteo Spedicato, Alberto Cavazzini, Martina Catani

**Affiliations:** 1 Department of Chemical, Pharmaceutical and Agricultural Sciences, University of Ferrara, via L. Borsari 46, 44121 Ferrara, Italy; 2 Department of Environmental and Prevention Sciences, University of Ferrara, via L. Borsari 46, 44121 Ferrara, Italy; 3 Department of Translational Medicine, University of Ferrara, via L. Borsari 46, 44121 Ferrara, Italy; 4 Department of Pharmacy, University of Medicine, Rruga e Dibrës 371, Tirana, Albania; 5 Council for Agricultural Research and Economics, CREA, via della Navicella 2/4, Rome, Italy

**Keywords:** *Hypericum perforatum*, Untargeted analysis, Reversed-phase liquid chromatography, Hydrophilic interaction liquid chromatography, High-resolution mass spectrometry

## Abstract

The achievement of a comprehensive profiling of plant metabolites has long represented a challenge, not only due to their wide-ranging abundances but also as a result of their considerable chemical diversity. Recent advances in highly sensitive liquid chromatographic (LC) techniques, particularly when coupled with high-resolution mass spectrometry (HRMS), have established metabolomics as a key approach for the analysis of thousands of non-volatile metabolites in crude natural extracts. Nevertheless, the different polarities of primary and secondary metabolites often limit the efficacy of conventional reversed-phase liquid chromatography (RPLC) in providing exhaustive compound coverage. To address this limitation, orthogonal separation techniques such as hydrophilic interaction liquid chromatography (HILIC) should be employed as a complement to RPLC. In this work, four columns with identical geometrical specifications but with different stationary phase chemistry (one reversed-phase C18 and three different HILIC adsorbents) were employed for the untargeted analysis of bioactive compounds contained in *Hypericum perforatum*. The columns were evaluated not only in terms of chromatographic performance but also based on their ability to resolve challenging isobaric compound pairs of isobaric compounds. Finally, the integration of RPLC and HILIC data enabled a more comprehensive characterization of the metabolites associated with the plant.

**Graphical Abstract:**

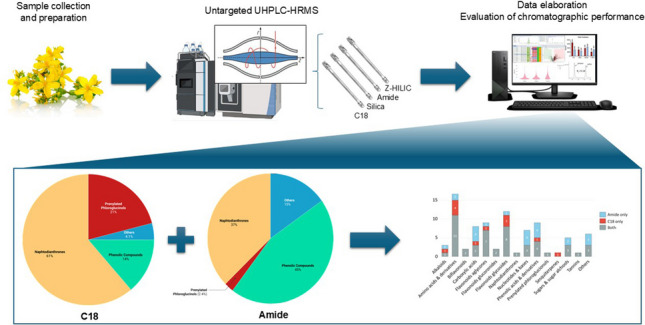

**Supplementary Information:**

The online version contains supplementary material available at 10.1007/s00216-025-06030-8.

## Introduction

Metabolomics is a high-throughput technique aimed at profiling low molecular weight metabolites present within a given biological system. Compared to other omics sciences (i.e. proteomics, transcriptomics and genomics), it provides a more detailed and comprehensive view of the phenotype of a biological system. For this reason, it is often considered the final step in the omics cascade.

Metabolomics analysis can be carried out either in targeted or untargeted modes. The targeted approach is typically adopted when a small set of well-known metabolites, often associated with the same metabolic pathway, needs to be determined and quantified. In contrast, the untargeted approach is aimed at detecting and annotating a large range of compounds within the sample. Consequently, targeted analysis can follow well-defined analytical procedures optimized for specific classes of metabolites, while untargeted metabolomics presents a greater challenge due to the significant chemical heterogeneity of the molecules involved. These may range from apolar to polar compounds, exhibiting diverse physio-chemical properties and spanning several orders of magnitude in concentrations.

Originally developed to analyse metabolites in biofluids, metabolomics has rapidly evolved into a fundamental tool for characterizing samples in different research areas, including environmental determinations, food science and characterization of natural extracts [1–7]. Among various biological models, plants are regarded as the most challenging systems due to the vast diversity of their metabolites. It is estimated that they may contain over 200,000 metabolites among primary and secondary ones, serving a wide range of functions from plant growth and development to the accumulation of specialized compounds in response to stress [8–11]. Consequently, plant metabolomics has emerged as a highly complex field, characterized by metabolites belonging to diverse chemical classes, including carbohydrates, amino acids, flavonoids, and alkaloids [12–14]. Since this is a recently emerged field, other limitations arise from the incomplete coverage of plant metabolites in spectral libraries. Even though specific databases covering different plant species have been recently introduced, many metabolites, including terpenoids and flavonoids, are still significantly underrepresented [15, 16]. As a result, only a small percentage of metabolites can be correctly annotated by comparison with high-quality experimental MS/MS spectra available in databases.

Ultra-high performance liquid chromatography ((U)HPLC) coupled with high-resolution mass spectrometry (HRMS) is one of the most widely used analytical techniques for the analysis of non-volatile metabolites. Reversed-phase liquid chromatography (RPLC) methods have long been favoured in metabolomics due to the wide range of available column chemistries and particle size, stability, and reproducibility in terms of retention times [17]. However, this approach alone often fails to provide full coverage of the metabolome, particularly when the sample contains a substantial number of polar or ionizable metabolites, as is common in plant extracts. To overcome this limitation, alternative separation techniques must be employed.

Hydrophilic interaction liquid chromatography (HILIC) represents one of the best approaches for the separation of polar analytes. This technique, introduced by Alpert in 1990 [18], relies on the use of polar stationary phases with eluents containing water and over 70% acetonitrile (ACN) with the addition of buffer salts. HILIC provides a very high degree of orthogonality with respect to RPLC; consequently, compounds with a strong retention in RPLC are typically poorly retained in HILIC, and vice versa [19, 20].

HILIC has gained significant popularity over the past years, not only due to its ACN-rich mobile phases that enhance compatibility with MS detection, but also as a result of the development of a wide array of adsorbents with diverse polarities and types of interactions (e.g. amide, diol, hydroxyethyl, sulfobetaine, zwitterions, etc.) [21]. Unlike RPLC, retention prediction in HILIC is still difficult, with selectivity being highly dependent on the chemistry of the stationary phase and the composition of the eluent. As a result, extensive screening of experimental conditions is often necessary to obtain adequate metabolite coverage. Despite this, most published studies on column comparison focus on targeted analyses, while only a limited number of studies have addressed untargeted metabolomics [22–25]. Therefore, a more comprehensive strategy must be adopted, typically involving separations in both positive and negative ionization modes using RPLC, alongside the application of orthogonal techniques such as HILIC.

To deepen this knowledge, in this work, a standard C18 column and three other HILIC stationary phases (namely silica, amide, and a zwitterionic one with sulfobetaine groups) have been employed to investigate naturally occurring bioactive compounds, particularly antioxidants, in *Hypericum perforatum* grown in the Balkan region. This medicinal herb, better known as St. John’s Wort (SJW), is widely recognized for its therapeutic potential in treating mood disorders, with global annual sales estimated in the billion dollars [26]. It is approved as a natural health product in Canada, classified as a dietary supplement in the USA, and listed as a medicinal herb by the European Medicines Agency [27–29].

To the best of our knowledge, this is the first study to thoroughly investigate the composition of SJW using untargeted metabolomics approaches and by employing HILIC adsorbents. The performance of the four chromatographic columns was compared, with a particular focus on understanding the differences in elution capabilities and selectivity for representative examples of isobaric compounds that are typically challenging to resolve using RPLC alone. The findings of this study may guide researchers in selecting optimal elution conditions for the untargeted characterization of antioxidant compounds, not only in SJW but also in other complex natural extracts.

## Materials and methods

### Samples, chemicals and reagents

*Hypericum perforatum* L. (SJW) plant aerial parts were collected in the mountain of Krraba, near Elbasan (Albania) during July 2023. They were transported to the Department of Pharmacy of the University of Medicine, Tirana, Albania, dried in a well-ventilated room, and separated from external impurities. Samples were then stored in a dark, dry place until analysis. LC–MS grade solvents (water (H_2_O), methanol (MeOH) and acetonitrile (ACN)) and additives (formic acid (FA) and ammonium formate) were from Carlo Erba Reagents (Milan, Italy). The pure standards of 20 proteinogenic amino acids and polyphenols, including gallic acid, luteolin, kaempferol, catechin, epicatechin, quercetin, isoquercitrin (Quercetin-3-O-glucoside), and rutin, as well as hypericin, hyperforin, uracil, and toluene were purchased from Merck Sigma-Aldrich (Darmstadt, Germany). Sodium carbonate, 2-diphenyl-1-picrylhydrazyl (DPPH), 6-hydroxy-2,5,7,8-tetramethylchroman-2-carboxylic acid (Trolox), and ethanol (EtOH) were also obtained from Merck Sigma-Aldrich.

### Columns and equipment

The chromatographic runs were carried out on a RP and three HILIC columns kindly provided by Waters**™** (Milford, MA, USA) with Ethylene Bridged Hybrid (BEH**™**) particle technology. Details of the employed columns are reported in Table 1.

**Table 1:** Geometrical and physico-chemical characteristics of the columns employed in the work as well as the acronyms that will be used in the manuscript to refer to the different columns

Column	Acronym	Type	Length (mm)	ID (mm)	Particle size (μm)	Pore size (Å)	Particle type
ACQUITY**™** Premier BEH**™** C18	C18	RPLC	100	2.1	1.7	130	C18
ACQUITY**™** Premier BEH**™** HILIC	Silica	HILIC	100	2.1	1.7	130	Bare silica
ACQUITY**™** Premier BEH**™** Amide	Amide	HILIC	100	2.1	1.7	130	Amide
Atlantis**™** Premier BEH**™** Z-HILIC	Z-HILIC	HILIC	100	2.1	1.7	95	Sulfobetaine

UHPLC-HRMS experiments were carried out on an UHPLC Vanquish Flex system (Thermo Fisher Scientific, Bremen, Germany), equipped with a binary pump, a thermostated autosampler, and a thermostated column compartment coupled with an Exploris 240 Q-Orbitrap spectrometer (Thermo Fisher Scientific) with a heated electrospray ionization (H-ESI) source.

Ultrasound-assisted extraction (UAE) was conducted in a DU-100 ultrasonic bath, manufactured by Giorgio Bormac (Carpi, Italy), featuring a temperature sensor and an ultrasonic power regulator. Absorbance measurements were performed using a Beckman DU730 UV–Vis spectrophotometer.

### Sample extraction

The extraction procedure was carried out using a combination of two extraction solvents and two extraction methods, resulting in a total of four tested combinations. The extraction solvents were mixtures of alcohol/water 80:20%(v/v) with either MeOH or EtOH, while the extraction techniques evaluated were ultrasound-assisted extraction (UAE) and magnetic stirring. Aerial parts of SJW were homogenized using a mortar and pestle prior to the extraction procedure. For each tested procedure, approximately 300 mg of homogenized sample was weighed and extracted with 3 mL of solvent for 30 min. Subsequently, the extracts were centrifuged at 5000 rpm for 5 min; then, the supernatant was collected. This procedure was repeated a total of 5 times for a total extraction volume of 15 mL. Throughout the extraction procedures, aluminium foil was used to minimize sample exposure to light, which is known to rapidly degrade some of SJW’s major components [30–33]. In the case of UAE, bath temperature was continuously monitored and maintained below 20 °C by the addition of ice, when necessary. Each extraction procedure was conducted in triplicate. The extracts were then stored in the dark at − 20 °C until further analysis. For all tested conditions, a blank was also prepared by adding the solvent for the same duration as the extraction, using the same laboratory consumables as those employed in the extraction procedure of the real sample.

### Total phenolic content (TPC)

Total phenolic content (TPC) assay was performed using a modification of the Folin-Ciocalteu method [34]. The procedure involved adding 500 µL of Folin-Ciocalteu reagent to 50 µL of SJW extracts and keeping the mixture in the dark for 5 min. At this point, 2 mL of Na₂CO₃ was added first, followed by the addition of water to reach a final volume of 10 mL. The solution was left at room temperature and in the dark for 90 min. Finally, absorbance was recorded at 700 nm. Results were obtained by comparison with a calibration curve constructed using gallic acid, ranging from 0.5 to 7.5 µg/mL (R^2^ = 0.9947) and expressed as mg of gallic acid equivalents per g of dried sample. Each of the three replicates per extraction condition was tested in duplicate.

### Radical scavenging activity (RSA)

The radical scavenging activity of SJW extracts was determined using a DPPH assay, according to the Fukumoto method [35] with some modifications. Briefly, 50 µL of extract was added to 1450 µL of DPPH solution. The absorbance was immediately measured (${t}_{0}$) at a wavelength of 515 nm. The samples were then stored in the dark for a total of 15 min and the absorbance was measured again (${t}_{1}$). The antioxidant activity was then evaluated as a percentage of DPPH inhibition at ${t}_{1}$ compared to ${t}_{0}$ by comparing the results with a calibration curve constructed with Trolox ranging from 0.05 mM to 0.075 mM (${R}^{2}$=0.9979). Final data was expressed in mMol of Trolox equivalents per g of dried sample. Each of the three replicates per extraction condition was tested in duplicate.

### UHPLC-ESI conditions

For each column, chromatographic conditions were kept as generic as possible, with minor adjustments based on the evaluation of total ion chromatograms (TIC) and extracted ion chromatograms (XIC) for some representative compounds available as standards. In this case, peak resolution and signal intensity were considered.

Extracts were filtered through 0.22 μm nylon filters and diluted 1:5 prior to injection into the UHPLC system. For elution on C18, the dilution was performed with water, whereas for HILIC columns it was done with ACN. For all tested columns, the injection volume was 1 µL and the flow rate was kept constant at 0.3 mL/min. Details regarding mobile phases and gradient conditions are provided in the Supplementary Information.

The ESI source parameters were optimized for positive and negative mode starting from those reported in [36] for the untargeted analysis of polyphenols. For positive ion mode, parameters were set as follows: the spray voltage was 3500 V, the sheath gas was set at 40 arbitrary units (a.u.), the auxiliary gas was set at 15 a.u., the sweep gas was set at 0 a.u., the ion transfer tube temperature was set to 300 °C, and the vaporizer temperature was set to 400 °C. For negative ion mode: the spray voltage was set at 2500 V, the sheath gas was set at 40 a.u., the auxiliary gas was set at 15 a.u., the sweep gas was set at 0 a.u., the ion transfer tube temperature was set to 350 °C, and the vaporizer temperature was set to 300 °C.

### HRMS data acquisition

The data were acquired in Data Dependent Acquisition (DDA) mode using the Acquire-X software (Thermo Fisher Scientific). Each sample was run in both positive and negative mode. Briefly, two different acquisition methods were created based on a full scan (FS) and a DDA method. Initially, the software performed a run with the FS method on a blank and on the sample to create an exclusion and an inclusion list that will be automatically added to the DDA method. Afterwards, three consecutive runs were performed on the sample using the DDA method that was automatically updated based on the precursors selected for fragmentation in the previous run. The FS acquisitions were also used for relative quantification; therefore, they were performed in triplicate. For both FS and DDA methods, the RF Lens were set to 70% and the scan range to 100–1000 m/z. For the FS method, the resolution was set to 180,000 full widths at half maximum at *m*/*z* 200 (FWHM), automatic gain control (ACG) target to 100% and maximum injection time to auto. For the DDA method, the full scan was performed at a resolution of 60,000 FWHM, ACG target to 100% and maximum injection time to auto, the DDA MS^2^ scans were performed with the cycle time mode set to 0.7 s with a resolution of 45,000 FWHM. The isolation window was 1.7 m/z, ACG target to 100% and maximum injection time to auto. The HCD normalized energy was 20, 50, 80 in positive mode and 20, 40, 60 in negative mode.

### Data processing and compound annotation

Data processing was performed using Compound Discoverer 3.3 software (Thermo Fisher Scientific). For each sample, the following data were loaded: a blank acquired with FS method to mark and remove compounds identified in the blank, 3 runs in FS mode for relative quantification, and 3 runs with DDA for compound identification.

Retention time alignment was performed using the ChromaAlign algorithm [37]. A mass tolerance of $\pm$ 5 ppm was used for the XIC creation of the detected features. Additionally, a peak rating filter, available within the software and based on the chromatographic characteristics of the XICs, was applied so that only features with a peak rating > 5 in at least one of the replicates were further processed.

Analytes were annotated using the MzCloud spectral library (MSI confidence level 2) and MzVault, the latter being implemented with data recorded from available standards (MSI level 1) [38].

After data processing, only features with a sample/blank ratio > 5 in all replicates were counted for the total feature count. For the count of features with MS^2^ data, only features with fragmentation data as preferred ions were considered. Finally, features were considered as matched with the spectral library only when the matching factor was above 85% with an annotated delta mass $\le$ 2 ppm.

Only the data regarding tailing factor and FWHM were obtained by processing the raw files with MZmine [39], using parameter settings as closely matched as possible to those applied during the main processing with Compound Discoverer.

## Results and discussion

The scope of this study was to evaluate the chromatographic performance and elution properties of four different adsorbents towards the untargeted analysis of a complex natural extract. To ensure broad applicability, the chromatographic methods were designed to be as general as possible by choosing commonly used mobile phases for RPLC and HILIC. A typical mass range for phenolic compound annotation was chosen, and it was experimentally confirmed that no significant signals were detected at m/z higher than 1000. MS resolution values were chosen to ensure a good spectral quality and to improve compound annotation, which is often limited by the scarce availability of spectral libraries for plant metabolites. Different gradient conditions were explored, and the separation has been optimized based on the quality of TIC signal and on the injection of pure analytical standards of compounds expected to be present in SJW. Notably, these compounds are characterized by different chemo-physical properties and, accordingly, they exhibit very different retention times. Prior to the chromatographic experiments and evaluating column performance, several extraction methods were qualitatively assessed to determine the most suitable approach, as detailed below.

### TPC and RSA results: optimization of extraction conditions

Dried SJW samples were extracted using a solid–liquid extraction procedure, as described in the “Sample extraction” section. Two extraction methods (magnetic stirring and UAE) were evaluated in combination with two hydroalcoholic extraction mixtures: MeOH/H_2_O and EtOH/H_2_O (80:20% v/v). These solvent mixtures are widely regarded as among the most effective to extract polyphenols from natural matrices [40, 41]. Qualitative spectrophotometric assays were used to select the most effective extraction procedure in terms of RSA and TPC.

The results of the spectrophotometric assays are reported in Table 2. Statistical significance was assessed using analysis of variance (ANOVA), followed by Tukey’s honestly significant difference (HSD) post hoc test (see Supplementary Information). The TPC values appeared to be more influenced by the choice of extraction solvent than by the extraction method, with methanolic extracts yielding the highest values. Among the tested procedures, MeOH UAE resulted in the extract with the highest TPC, whose value was significantly higher (*p* < 0.05) than that of both ethanol extracts but not significantly different from MeOH stirred.

**Table 2:** Main results and standard deviation of TPC and RSA assays on the four SJW extracts

	Total phenolic content (TPC) (Eq gallic acid (mg)/dw (g))	Dev. Std (±)	Radical scavenging activity (RSA) (µMol eq Trolox/g dw)	Dev. Std (±)
MeOH stirred	68.3	2.3	348	25
EtOH stirred	51.2	1.7	251	45
MeOH UAE	73.9	6.3	477	56
EtOH UAE	61.5	5.6	374	35

With regards to RSA, extraction efficiency appeared to be more affected by the extraction method than the solvent, with UAE providing better yields. However, also in this case, MeOH UAE resulted in the highest RSA. The RSA of the MeOH UAE extract was significantly higher (*p* < 0.05) than that of EtOH stirred and MeOH stirred but not significantly different from EtOH UAE.

Overall, these results indicate a clear trend, allowing us to identify MeOH UAE as the most effective extraction procedure. Consequently, this method was selected for subsequent analysis.

### Chromatographic methods and features count

The chromatographic method development was carried out separately for the different columns by injecting both a mixture of analytical standards and the samples extracted in MeOH UAE. For the HILIC mode, it was found that using a mobile phase with a water content below 10% (v/v) had a negligible effect on increasing retention, particularly for less retained compounds, while significantly deteriorating chromatographic performance in terms of efficiency. For this reason, the initial mobile phase composition for all HILIC columns was set at 10% (v/v) water. Notably, the tested HILIC columns demonstrated good reproducibility throughout the experiments and maintained an equilibration time comparable to that of RPLC columns. To ensure comparability of results, the H-ESI source parameters were kept constant across all chromatographic runs.

Most untargeted approaches for the characterization of complex matrices aim to putatively identify as many compounds as possible. To achieve this, it is crucial to develop a LC-HRMS method capable of detecting the highest possible number of features. Secondly, compound annotation is generally performed by comparing experimentally obtained MS^2^ spectra with those available in commercial libraries. For this process to be effective, it is essential not only that features are detected but also that they are associated with at least one MS^2^ spectrum in a database or a library. Based on these considerations, the different chromatographic columns were compared by evaluating the total number of detected features, the number of features associated with an MS^2^ spectrum, and the number of these with a high spectral match with commercial library (85% was chosen as a threshold).

**Fig. 1:**
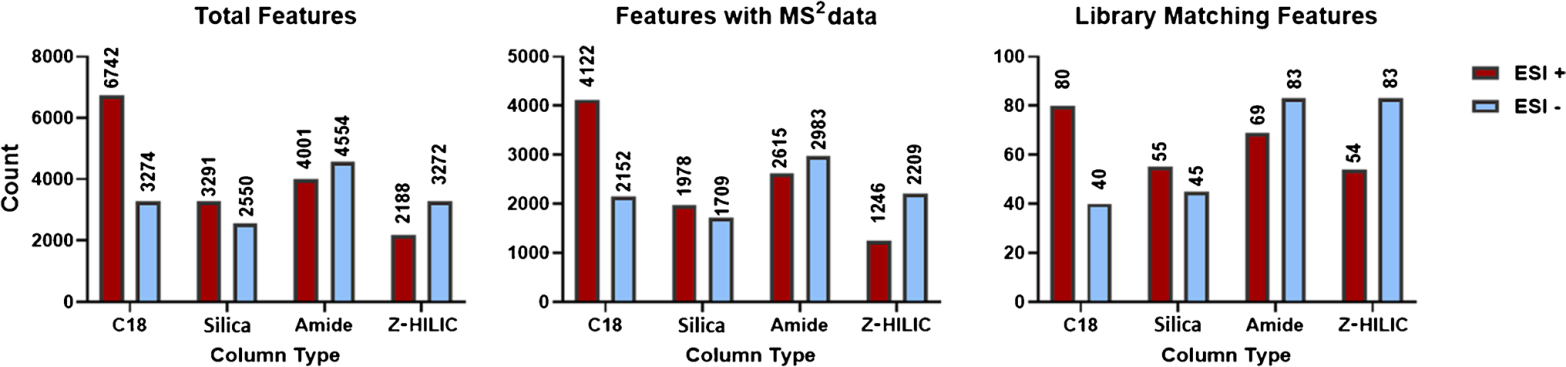
Bar charts showing the number of total features (left), number of features with MS^2^ data (centre) and library matching features (right) for the four surface chemistry types (BEH C18, BEH HILIC, BEH Amide, and BEH Z-HILIC) in positive (red) and negative (blue) ionization modes

Across both positive and negative ionization modes, the C18 column produced the highest total feature count, especially in positive mode over 6700 features (Fig. 1). However, the differences become less pronounced when considering features with MS^2^ data and, to an even greater extent, those with a library match. This may reflect the enhanced separation of polar isobaric compounds (e.g. sugars or phenolic acids) by HILIC columns, which co-elute near the void time in RPLC and are thus detected as a single feature.

By restricting the comparison only to HILIC columns in Fig. 1, the amide stationary phase demonstrated the best performance across all evaluated parameters, particularly in negative mode. Nevertheless, it is important to emphasize that while the total number of detected features can be useful as a preliminary screening method evaluation, a higher feature count does not automatically indicate superior method performance. This is due to the possible presence of non-matrix-related features, such as contaminants and instrumental noise [42, 43]. Moreover, the number of spectral matches is strongly influenced by the composition and comprehensiveness of compounds included in the spectral library employed. In this regard, it is well known that the coverage of plant-derived metabolites in available libraries represents a significant limiting factor, often reducing the number of reliable matches obtained [44].

### Chromatographic performance

Efficiency plays a crucial role in column selection. Narrow elution peaks increase the analyte concentration over a shorter time window, leading to improved sensitivity. In contrast, broad chromatographic peaks significantly increase the probability of co-elutions, which can result in ion suppression effects and a decrease in ionization efficiency. Additionally, broad peaks can make correct integration for quantitative purposes particularly challenging when isobaric compounds are present. In extreme cases, when peaks are excessively broad, data processing algorithms may misinterpret a single eluting analyte as multiple distinct features.

For these reasons, the efficiency of the tested columns was evaluated in terms of band broadening by extrapolating full width at half maximum (FWHM) values for all detected features. Since all the columns share the same physical dimensions (length and I.D.), packing technology, and particle size, the observed differences in peak broadening can be primarily attributed to the chemistry of their stationary phases and retention mechanisms.

The results reported in Table 3 show the percentage of detected features (relative to the total number for each column and ESI mode) with FWHM values below 0.1, 0.075, and 0.05 min. These data clearly indicate that, in terms of chromatographic efficiency, the C18 column outperformed the HILIC columns.

**Table 3:** Percentage of features, out of the total detected for each column and ESI mode (+ and -) and for each surface chemistry (BEH C18, BEH HILIC, BEH Amide, and BEH Z-HILIC), with an FWHM lower than the reported value

	C18 +	C18 -	Silica +	Silica -	Amide +	Amide -	Z-HILIC +	Z-HILIC -
< 0.1 min	78.3	80.7	65.6	65.5	63.8	66.2	48.8	42.8
< 0.075 min	62.6	68.5	52.8	51.8	45.8	47.9	34.5	30.4
< 0.05 min	25.2	29.2	23	20.3	19.9	21.2	14.1	11.7

**Fig. 2:**
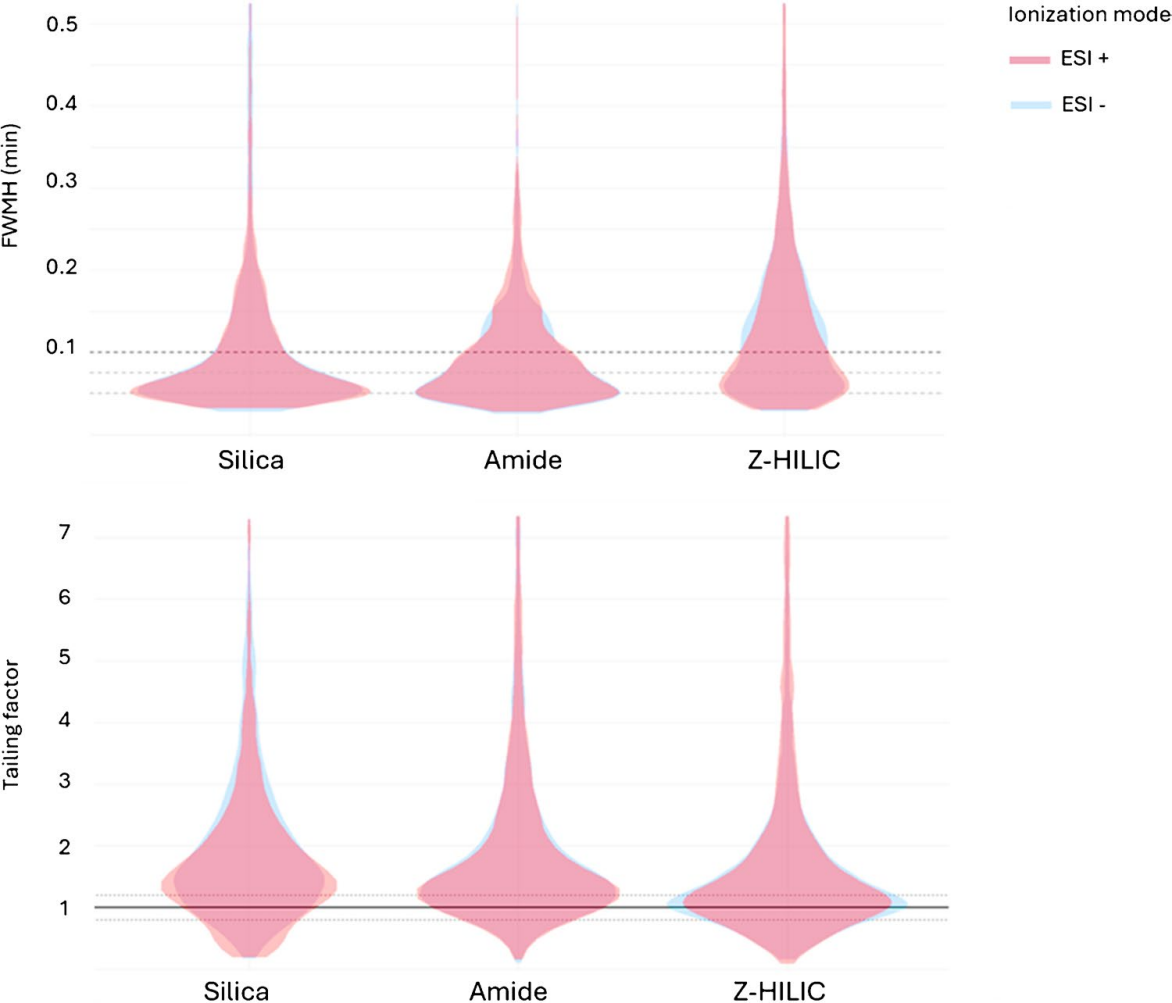
Violin plots showing the frequency distribution of full width at half height (FWMH) in min (top) and tailing factor (bottom) on the x-axis for each detected feature on the three HILIC surface chemistry types (BEH HILIC, BEH Amide, and BEH Z-HILIC) in positive (red) and negative (blue) ionization modes

To better visualize the differences among the HILIC adsorbents, the C18 was excluded from the analysis shown in Fig. 2 (top) panel. From the visualization of FWHM distributions by means of violin plots, it is evident that the bare silica column achieved the highest efficiency among the HILIC options. Its FWHM distribution was particularly narrow and concentrated at low values, with approximately 35% of features falling within 0.05 ± 0.01 min and about 65% below 0.1 min. The amide column showed a similar trend, though with slightly broader peaks. In contrast, the Z-HILIC stationary phase performed significantly worse. No significant differences were observed between the results obtained in ESI + and ESI − modes for any of the columns tested.

### Peak symmetry

Peak symmetry is a critical parameter in evaluating the performance of chromatographic separations, as asymmetrical peaks, whether due to fronting or tailing, can compromise integration reproducibility. This issue is particularly relevant in untargeted LC-HRMS analysis, where due to the large volume of data, automated peak integration is needed. However, the algorithms used for peak integration often struggle with peaks that deviate from a gaussian shape, leading to potential quantification errors and inaccurate retention time assignments. These inaccuracies may consequently compromise peak alignment, which is a key step in untargeted workflows.

Peak symmetry was evaluated by calculating the tailing factor (TF), where a value of TF equal to one indicates ideal Gaussian symmetry. TF distributions were analysed for all detected features for each HILIC adsorbent and ionization mode.

The Z-HILIC stationary phase exhibited the best peak symmetry, with 28.4% of the features detected in ESI + and 30.8% in ESI − falling within the optimal TF range of 0.9 and 1.1 (Fig. 2, bottom). Interestingly, the bare silica column, despite the high efficiency, exhibited the poorest peak symmetry. These results highlight a complex relationship between peak tailing and column efficiency and that poor peak shape does not necessarily correlate with peak broadening and reduced efficiency.

### Retention behaviour

As previously discussed, co-elution represents a major challenge in untargeted metabolomics. Depending on their stationary phase, different chromatographic columns exhibit distinct retention properties, yet certain compound classes show minimal or no retention, eluting at or near the dead time. As a result, this chromatographic region is particularly prone to co-elution phenomena.

To evaluate this effect across the different columns, as well as the varying distribution of features within the separation space, the apparent retention factor ($k_{app}$) was calculated for each detected feature through the following equation:

$${k}_{app}=\frac{{t}_{R}-{t}_{0}}{{t}_{0}}$$

where $t_R$ is the retention time and $t_0$ the column dead time, evaluated by injecting uracil in RPLC and toluene in HILIC mode as dead time markers.

**Fig. 3:**
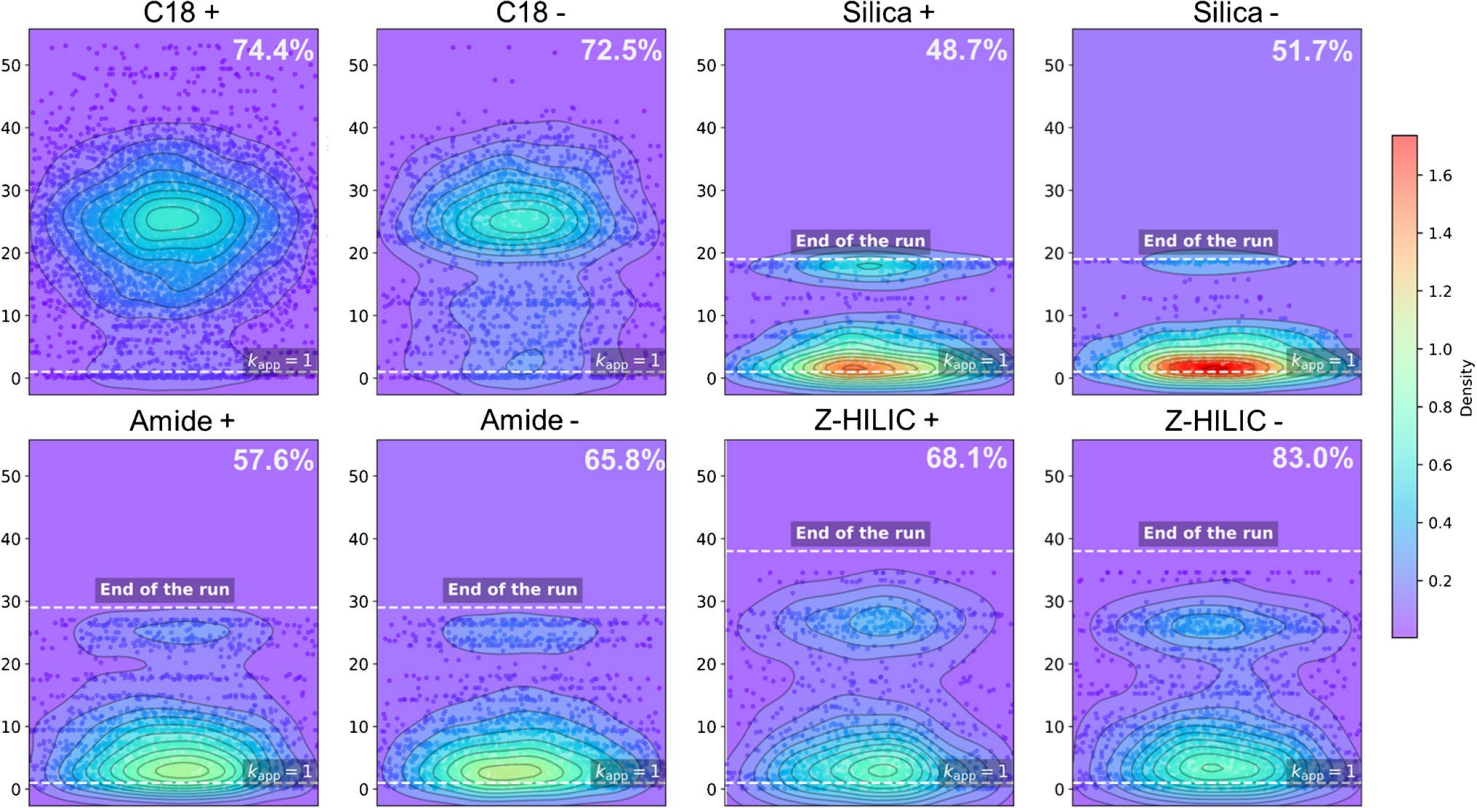
Contour plots showing the distribution of $k_{app}$ values (y-axes) for each detected feature on the four surface chemistry types for the surface chemistry types (BEH C18, BEH HILIC, BEH Amide, and BEH Z-HILIC) in both positive and negative ionization modes. In the top right corner of each contour plot the percentage of features with $k_{app}$ ≥ 1 is reported, calculated over the total number of detected features with the corresponding combination of columns and ionization mode

The distribution of retention factors is presented by means of contour plot (Fig. 3). Additionally, the percentage of features with ${\text{k}}_{\text{app}}$ ≥ 1, used as an indicative threshold for acceptable retention [45], was determined for each column and ionization mode. As expected, the C18 column provided an almost uniform distribution within the separation space. The HILIC columns, on the other hand, exhibited different trends with respect to the C18. The bare silica column showed very low retention, with nearly half of the detected features eluting at or near the dead time. This may explain its high efficiency but poor peak symmetry. Experimental findings suggest a significantly different scenario with the amide column and even more with the Z-HILIC, for which almost 83% of the detected features in ESI − showed ${\text{k}}_{\text{app}}$ ≥ 1. This result aligns with previous reports on this column, which is known to exhibit higher retention, compared to other HILIC columns, due to the chemistry of the stationary phase which enables interactions with both anions and cations. Additionally, the smaller pore size of its packing material could increase the available interaction surface [46].

Interestingly, both the amide and Z-HILIC columns exhibited a pronounced bimodal retention pattern, with analytes either poorly or strongly retained, with an almost empty intermediate region. This pattern contrasts with the more homogeneous distribution observed for the C18 column, where retention factors are more evenly spread across the separation space.

### Resolution of critical pairs of compounds

Plant matrices are rich in isobaric metabolites of different chemical nature. While some of these compounds can be distinguished by their MS^2^ spectra, this is often challenging due to highly similar or even identical fragmentation patterns. For this reason, in LC-HRMS analyses, it is crucial to achieve a good baseline separation to ensure accurate identification and quantification. To assess the capability of the tested columns to separate critical pairs of compounds, the chromatographic resolution (${R}_{s}$) was calculated for four pairs of isobaric molecules contained in SJW through the following equation:

$${R}_{s}=\frac{2({t}_{R,2}-{t}_{R,1})}{{w}_{b,1}+{w}_{b,2}}$$

where ${t}_{R,2}$ and ${t}_{R,1}$ are the retention times of the most retained and least retained analytes, respectively, and ${w}_{b}$ represents the baseline peak widths.

**Fig. 4:**
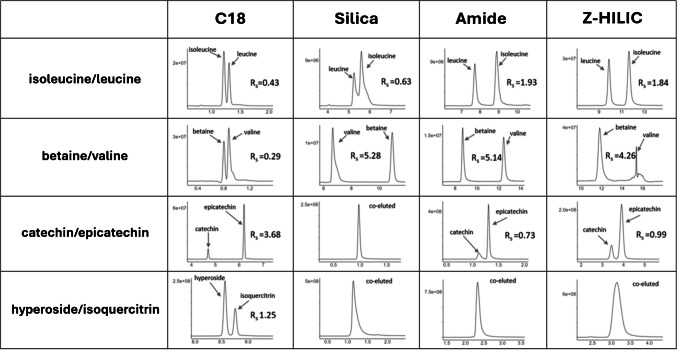
Experimental profiles of critical pairs of compounds on the four surface chemistry types (BEH C18, BEH HILIC, BEH Amide, and BEH Z-HILIC)

The selected compounds were chosen based on their biological relevance and the known challenges associated with their separation using conventional techniques. At least one compound for each pair was confirmed using pure standards, based on both retention time and fragmentation spectra. Extracted ion chromatograms (XICs) and calculated resolution values for all four tested columns are shown in Fig. 4. Notably, these results were obtained using a generic chromatographic method commonly employed in untargeted workflows. In cases of partial co-elution, the peak width at the baseline was estimated by projecting the peak shape down to the baseline, assuming a Gaussian-like profile.

Leucine and isoleucine are structural isomers with very similar physico-chemical properties which make them particularly challenging to be separated [47]. Furthermore, not only do they exhibit very poor retention on C18 columns due to their high polarity but also, they cannot be distinguished based on their fragmentation spectra obtained via higher-energy collisional dissociation (HCD). As expected, the resolution of this pair on the C18 column was suboptimal $R_s=0.43$. Separation was insufficient also on the bare silica column $(R_s=0.63)$, whereas both the amide and Z-HILIC columns showed baseline separation. It must be pointed out that the formula used to calculate resolution considers only geometric parameters such as relative distance and peak width, without accounting for peak shape, asymmetry, or visual overlap. In the case of narrow and symmetric peaks, such as the leucine-isoleucine pair on the C18 column, a minimal separation might appear visually acceptable but is strongly penalized in the numerical calculation of resolution due to the very small difference in retention times.

Betaine and valine are constitutional isomers, presenting similar separation challenges as the leucine-isoleucine pair. However, unlike leucine and isoleucine, betaine and valine can be differentiated by their HCD fragmentation spectra but also for this pair separation in C18 was inadequate. Conversely, all three HILIC columns provided excellent resolution. However, it is worth mentioning that on the Z-HILIC column, a strong ion suppression effect was observed for valine. A possible explanation for this phenomenon could be its co-elution with the sodium formate cluster [Na(HCOONa)_3_]^+^, which is known to cause significant ion suppression [48]. Under the applied chromatographic conditions, the elution of this cluster occurred in the region with the highest feature density (see previous section), suggesting that the Z-HILIC column may be a suboptimal choice, particularly in ESI + mode.

Catechin and epicatechin are among the most studied polyphenols due to their antioxidant properties, for instance, in tea leaves (49). These molecules, which are diastereoisomers, are considerably less polar than the previously discussed analytes, therefore they were well retained and effectively separated in C18. On the HILIC columns, the bare silica column showed complete co-elution, the amide column achieved partial were separation $\left(R_s=0.73\right)$. The Z-HILIC column provided a good separation approaching baseline resolution $\left(R_s=0.99\right)$. 

Finally, hyperoside and isoquercitrin differ in the type of sugar moiety linked at position 3 of the quercetin aglycone, with isoquercitrin being the 3-O-glucoside of quercetin and hyperoside being the 3-O-galactoside of quercetin. In this case, none of the tested HILIC columns provided even partial separation, as the two analytes were completely co-eluted in the experimental conditions used in this work. On the other hand, the C18 column showed acceptable separation (${R}_{s}$= 1.25).

### Characterization of SJW extracts by combining RPLC and HILIC

Based on the findings observed through column evaluation and by considering its strong performance across all evaluated parameters (number of putative compounds identified, chromatographic performance, and resolution of critical pairs of isobaric compounds), the amide column was selected to complement the C18 column for a more comprehensive characterization of the hydroalcoholic SJW extract. All putative identified compounds from both C18 and the amide columns were manually reviewed to eliminate ambiguous assignments and to remove misinterpreted features generated by the data processing software. The complete list of identified and tentatively identified compounds, along with the corresponding details, is provided in Supporting Information. For both columns, data obtained in positive and negative ionization modes were merged.

**Fig. 5:**
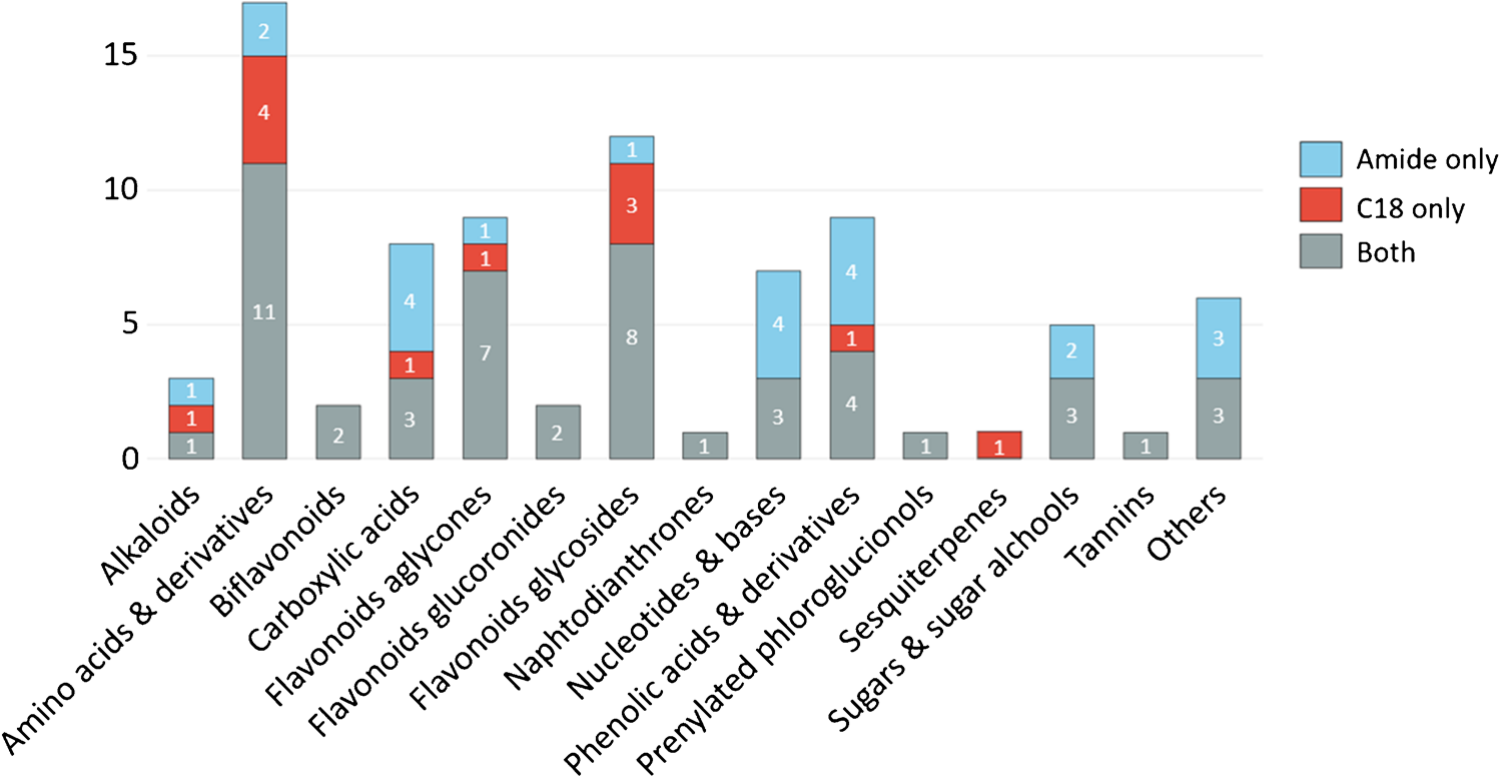
Bar chart illustrating the number of putatively identified compounds per molecular class detected exclusively on the BEH C18 column (red), exclusively on the BEH Amide column (light blue), or on both columns (grey)

Overall, the C18 column led to the tentative identification of 62 compounds (ID level = 2), of which 25 were confirmed by comparison with authentic standards and thus classified as confidently identified (ID level = 1). The amide column enabled the putative identification of 72 compounds, with 15 of these confirmed at ID level = 1. Among the annotated compounds, 50 were detected on both columns, while 12 were uniquely identified on the C18 column and 22 were exclusive to the amide column (Fig. 5). Notably, the amide column expanded the identification of acidic and basic compounds, detecting 8 additional carboxylic and phenolic acids as well as 4 nucleobases that were not observed on the C18 column. Conversely, the C18 column facilitated the identification of a higher number of amino acids (and their derivatives) and flavonoid glycosides.

## Conclusions

The selection of the most suitable analytical column is crucial for untargeted metabolomics approaches, particularly when analysing complex natural matrices containing chemically diverse compound classes. Therefore, the chemo-physical properties of the stationary phase critically impact both the number and the nature of putative identified compounds.

The chromatographic performance of the columns considered in this work indicated that the C18 column outperformed all tested HILIC ones, delivering the best overall performance. Consequently, RPLC can be well regarded as a highly effective option for initial untargeted approaches, at least on natural matrices similar to SJW. However, relying only on RPLC may result in the loss of valuable information. Therefore, complementary techniques, particularly HILIC, can enhance metabolome coverage. Among the tested HILIC columns, the amide one showed the best balance in terms of kinetic performance and retention properties. Nonetheless, the Z-HILIC column also showed promising characteristics, thus making it a valuable choice to achieve complementary results with respect to RPLC. On the other hand, the bare silica column showed insufficient retention for most of the compounds contained in the matrix.

The findings of this work suggest that to achieve optimal results researchers should test and tailor the analytical method to each specific matrix and set of analytes under investigation. Moreover, a single chromatographic column may not provide a complete snapshot of the full chemical composition of complex natural extracts. Therefore, employing different adsorbents and chromatographic conditions is necessary to achieve a more complete fingerprinting of the sample. This approach inevitably translates into additional time and effort; however, the employment of comprehensive multidimensional techniques, when available, can significantly enhance the depth of investigation within a single chromatographic run. Lastly, it is important to note that the identification of plant-derived metabolites remains limited by the incomplete coverage of compounds in spectral libraries, which often leads to many putative annotations. However, using complementary elution methods can increase the confidence of the identification through the comparison of retention behaviour across different stationary phases.

## Supplementary Information

Below is the link to the electronic supplementary material.

Supplementary Material 1 (PDF 551 KB)

## Data Availability

All data are included in the paper and in the Supporting Information.
