# Supplementary material for: Comparative evaluation of reversed-phase and hydrophilic interaction liquid chromatography columns for untargeted profiling of bioactive compounds in Hypericum perforatum
Source: Anal Bioanal Chem. 2025 Jul 26;418(2):687–99. doi: 10.1007/s00216-025-06030-8 (PMC12783305; doi:10.1007/s00216-025-06030-8)
Supplement: Supplementary file 1 — Supplementary Material 1 (PDF 551 KB) [file 216_2025_6030_MOESM1_ESM.pdf]

SUPPLEMENTARY INFORMATION

Comparative Evaluation of Reversed-Phase and Hydrophilic Interaction Liquid Chromatography Columns for Untargeted Profiling of Bioactive Compounds in *Hypericum perforatum*

Davide Barboni[a], Desiree Bozza[a], Damiana Natasha Spadafora[b], Nicoletta Bianchi[c], Brunilda Myftari[d], Paola Tedeschi[a], Chiara De Luca[a], Simona Felletti[b], Matteo Spedicato[a], Alberto Cavazzini[a,e], Martina Catani[a,*]

[a]*Department of Chemical, Pharmaceutical and Agricultural Sciences, University of Ferrara, via L. Borsari 46, 44121, Ferrara, Italy*

[b]*Department of Environmental and Prevention Sciences, University of Ferrara, via L. Borsari 46, 44121, Ferrara, Italy*

[c]*Department of Translational Medicine, University of Ferrara, via L. Borsari 46, 44121, Ferrara, Italy*

[d]*Department of Pharmacy, University of Medicine, Rruga e Dibrës 371, Tirana, Albania*

[e]*Council for Agricultural Research and Economics, CREA, via della Navicella 2/4, Rome, Italy*

*Corresponding author: *martina.catani@unife.it*

1. Chromatographic conditions

C18: The extracts were filtered through 0.22 µm nylon filters and diluted 1:5 with water. Injection volume was 1 µl and the flow rate was set at 300 µl/min. Mobile phases consisted of H_2O + 0.1 % (v/v) FA (phase A) and ACN + 0.1% (v/v) FA (phase B). The gradient was as follows: 5% B isocratic elution for 0.5 min, 5–30% phase B in 12.5 min, 30–80% phase B in 3 min; 80-98 % phase B in 10 min, 98% B was held for 11 min, followed by a 4 min re-equilibration at 5% B. Throughout the experiments, the column temperature was held constant at 30°C.

Silica: Extracts were filtered through 0.22 µm nylon filters and diluted 1:5 with ACN. The injection volume was 1 µl and the flow rate was 300 µl/min. The column compartment temperature was maintained at 25 °C. The mobile phases were: 10 mM ammonium formate + 0.1% (v/v) FA (phase A) and 90:10 % ACN/Phase A (Phase B). The gradient was set as follows: 100% B was held for 10 min, 100-60% B in 1 min and held for 3 minutes 100% B was restored and held for 4 minutes for re-equilibration.

Amide: Extracts were filtered through 0.22 µm nylon filters and diluted 1:5 with ACN. The injection volume was 1 µl and the flow rate was 300 µl/min. The column compartment temperature was maintained at 25 °C. The mobile phases were 10 mM ammonium formate + 0.1% (v/v) FA (phase A) and 90:10 % ACN/Phase A (Phase B). The gradient was set as follows: 100% B was held for 10 min, 100-60% B in 1 min and held for 3 minutes 100% B was restored and held for 4 minutes for re-equilibration. The gradient was: 100% B for 12 min, 100-60% in 5 min, 60% held for 4 min followed by a 4 min re-equilibration at 100% B.

Z-HILIC: Extracts were filtered through 0.22 µm nylon filters and diluted 1:5 with ACN. The injection volume was 1 µl and the flow rate was 300 µl/min. The column compartment temperature was maintained at 25 °C. The mobile phases were 10 mM ammonium formate + 0.1% (v/v) FA (phase A) and 90:10 % ACN/Phase A (Phase B). The gradient was set as follows: 100% B was held for 10 min, 100-60% B in 1 min and held for 3 minutes 100% B was restored and held for 4 minutes for re-equilibration. The gradient was: 100% B for 12 min, 100-60% in 5 min, 60% held for 4 min followed by a 4 min re-equilibration at 100% B.

2. Statistic evaluation of results of the TPC test

	MeOH UAE	MeOH stirred	EtOH UAE	EtOH stirred
MeOH UAE	-	0.48	1.38	2.89
MeOH stirred	0.48	-	0.32	2.13
EtOH UAE	1.38	0.32	-	1.05
EtOH stirred	2.89	2.13	1.05	-

Table S1*: Post-Hoc Tukey test results on the TPC values expressed as -logP for each couple of extracts.*

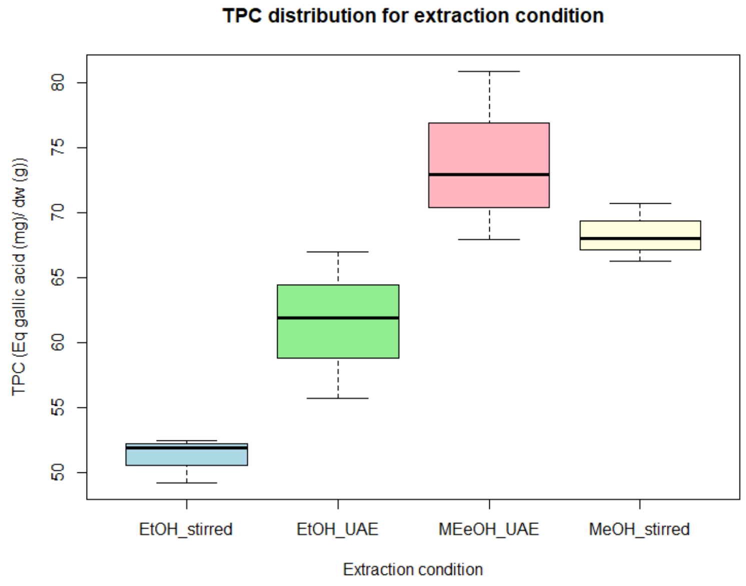

Figure S1: *Box plot showing the TPC of each extract in eq. of gallic acid (mg) for each g of dry weight.*

3. Statistic evaluation of results of the RSA test

	MeOH UAE	MeOH stirred	EtOH UAE	EtOH stirred
MeOH UAE	-	0.06	1.18	3.11
MeOH stirred	0.06	-	1.64	1.08
EtOH UAE	1.18	1.64	-	1.54
EtOH stirred	3.11	1.08	1.54	-

***Table S2**: Post-Hoc Tukey test results on the RSA values expressed as -logP for each couple of extracts.*

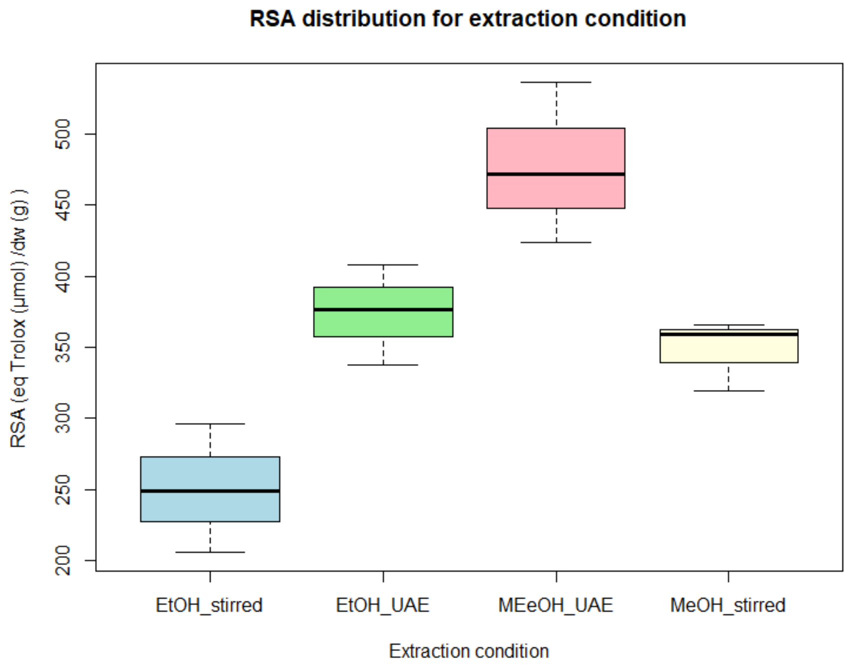

***Figure S2:** Box plot showing the TPC of each extract in eq. of gallic acid (mg) for each g of dry weight.*

4. Pie Charts

The following pie charts report the coverage of metabolite classes for both the C18 (figure S3) and the amide (Figure S4) columns.

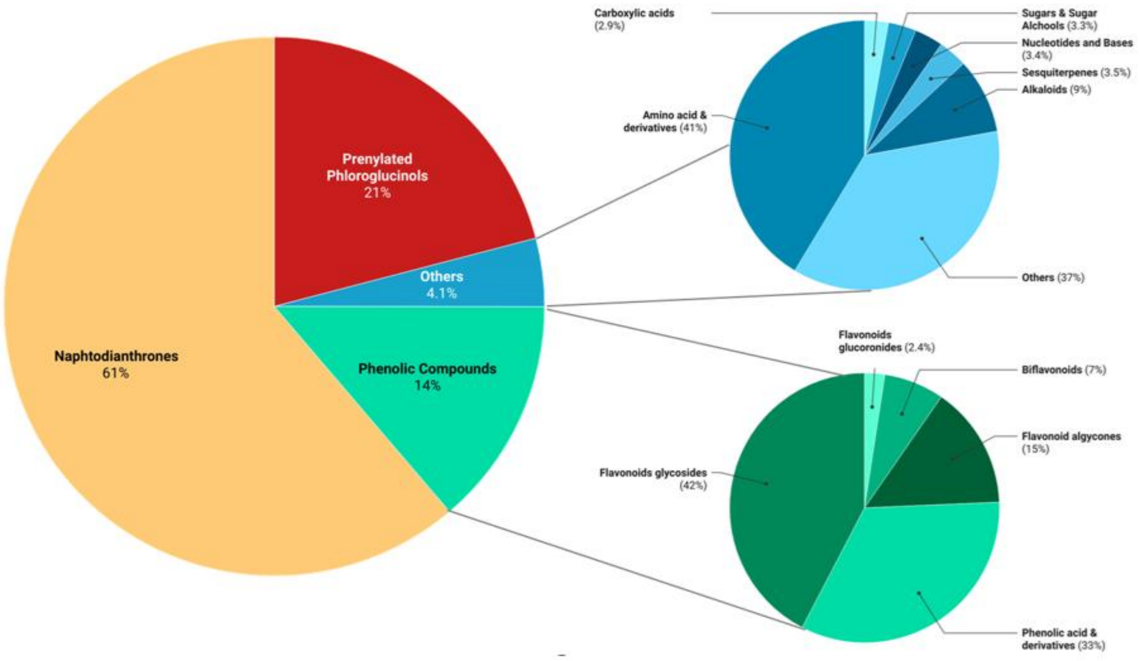

Figure S3: *pie chart reporting the coverage of metabolite classes according to results obtained on the C18 column.*

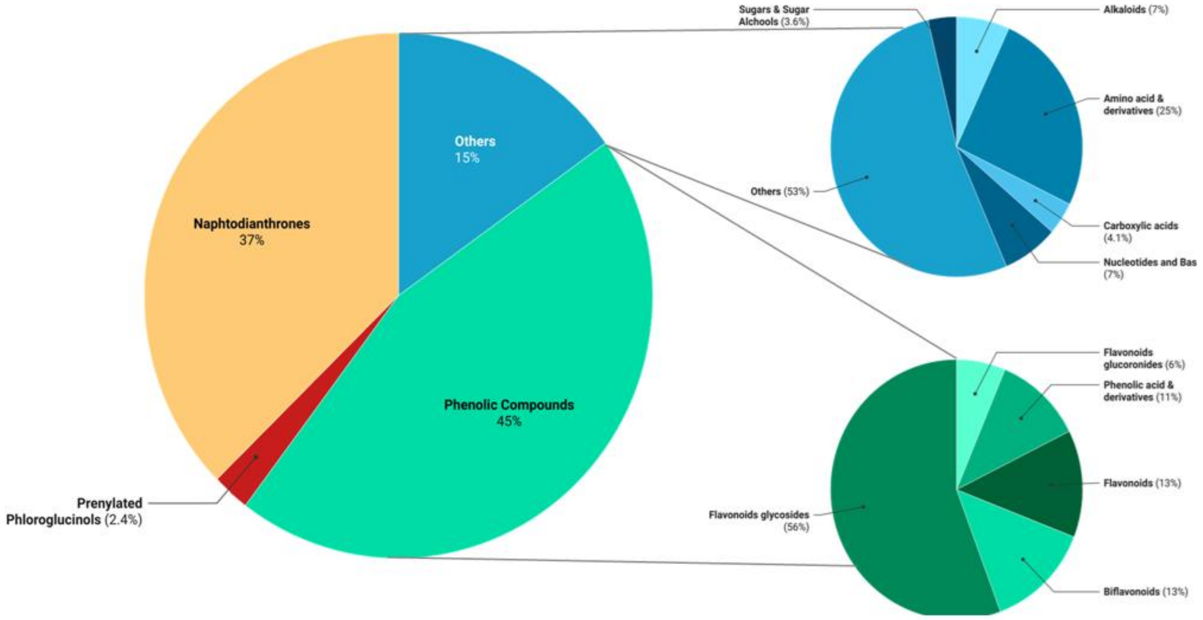

Figure S4: *pie chart reporting the coverage of metabolite classes according to results obtained on the amide column.*

5. Putative compounds

The following tables report the details of putative compounds identified on the C18 (Table S3) and amide (Table S4) columns.

Name	Class	Formula	Δ Mass [ppm]	Calc. MW	m/z	RT [min]	Reference Ion	MzCloud match	Area Average	ID level
Stachydrine	Alkaloids	C7 H13 N O2	-0,96	143,09449	144,10177	0,827	$[M+H]^{+1}$	97,8	7,46E+06	2
Trigonelline	Alkaloids	C7 H7 N O2	-1,15	137,04752	138,0548	0,802	$[M+H]^{+1}$	99,9	1,63E+08	2
Arginine	Amino acid & derivatives	C6 H14 N4 O2	-1,14	174,11148	175,11875	0,722	$[M+H]^{+1}$	99,2	3,03E+07	2
Glutamic acid	Amino acid & derivatives	C5 H9 N O4	-1,08	147,053	148,06031	0,779	$[M+H]^{+1}$	97,6	1,95E+07	1
Histidine	Amino acid & derivatives	C6 H9 N3 O2	-0,9	155,06934	156,07661	0,718	$[M+H]^{+1}$	95,4	5,21E+06	1
Isoleucine	Amino acid & derivatives	C6 H13 N O2	-0,98	131,0945	132,10178	1,235	$[M+H]^{+1}$	99,6	6,46E+07	1
Kynurenic acid	Amino acid & derivatives	C10 H7 N O3	-1,04	189,0424	190,04967	4,2	$[M+H]^{+1}$	98,8	3,26E+06	2
Leucine	Amino acid & derivatives	C6 H13 N O2	-1,14	131,09448	132,10176	1,334	$[M+H]^{+1}$	99,5	5,34E+07	1
Lysine	Amino acid & derivatives	C6 H14 N2 O2	-0,64	146,10543	147,11271	0,715	$[M+H]^{+1}$	98,5	9,37E+06	1
Phenylalanine	Amino acid & derivatives	C9 H11 N O2	-1,06	165,0788	166,08608	2,021	$[M+H]^{+1}$	95	6,73E+07	1
Proline	Amino acid & derivatives	C5 H9 N O2	-1,55	115,06315	116,07043	0,815	$[M+H]^{+1}$	99,8	3,29E+08	1
Prolylleucine	Amino acid & derivatives	C11 H20 N2 O3	-0,99	228,14717	229,15444	1,308	$[M+H]^{+1}$	93	5,68E+06	2
Serine	Amino acid & derivatives	C3 H7 N O3	-1,61	105,04242	106,0497	0,769	$[M+H]^{+1}$	91,4	9,60E+06	1
Threonine	Amino acid & derivatives	C4 H9 N O3	-1,24	119,0581	120,06537	0,779	$[M+H]^{+1}$	96,7	1,49E+07	1
Tryptophan	Amino acid & derivatives	C11 H12 N2 O2	-0,9	204,08969	205,09697	3,73	$[M+H]^{+1}$	99,5	3,18E+07	1
Tyrosine	Amino acid & derivatives	C9 H11 N O3	-0,99	181,07371	182,08099	1,116	$[M+H]^{+1}$	98,8	2,20E+07	1
Valine	Amino acid & derivatives	C5 H11 N O2	-1,23	117,07883	118,08611	0,881	$[M+H]^{+1}$	99	1,14E+08	1
Biapigenin (Amentoflavone)	Biflavonoids	C30 H18 O10	0,65	538,09035	537,08303	15,243	$[M-H]^{-1}$	95,6	4,08E+08	2
Biapigenin isomer	Biflavonoids	C30 H18 O10	1,32	538,09071	537,08343	15,45	$[M-H]^{-1}$	92,6	4,97E+07	2
Glyceric Acid	Carboxylic acids	C3 H6 O4	0,95	106,02671	105,01943	0,842	$[M-H]^{-1}$	92,6	2,90E+06	1
Guanidinobutyric acid	Carboxylic acids	C5 H11 N3 O2	-0,69	145,08503	146,0923	0,87	$[M+H]^{+1}$	97	6,66E+06	2
Indoleacrylic acid	Carboxylic acids	C11 H9 N O2	-1,26	187,06309	188,07043	3,731	$[M+H]^{+1}$	91,3	2,08E+07	2
Malic acid	Carboxylic acids	C4 H6 O5	0,76	134,02163	133,01435	0,946	$[M-H]^{-1}$	95,3	2,36E+07	2
Apigenin	Flavonoids	C15 H10 O5	0,77	270,05303	269,04575	16,781	$[M-H]^{-1}$	96,1	1,76E+07	2
Catechin	Flavonoids	C15 H14 O6	1,01	290,07933	289,07205	4,701	$[M-H]^{-1}$	95,2	4,29E+07	1
Diosmetin	Flavonoids	C16 H12 O6	-1,04	300,06308	301,07035	14,936	$[M+H]^{+1}$	92,9	1,08E+06	2
Epicatechin	Flavonoids	C15 H14 O6	0,55	290,0792	289,07192	6,199	$[M-H]^{-1}$	95,3	2,19E+08	1
Keampferol	Flavonoids	C15 H10 O6	0,91	286,048	285,04072	14,938	$[M-H]^{-1}$	96,9	1,83E+07	1
Luteolin	Flavonoids	C15 H10 O6	0,91	286,048	285,04072	12,935	$[M-H]^{-1}$	92,8	8,50E+06	1
Quercetin	Flavonoids	C15 H10 O7	0,29	302,04274	301,03545	12,988	$[M-H]^{-1}$	99,6	4,09E+08	1
Taxifolin	Flavonoids	C15 H12 O7	-1,19	304,05794	305,06522	9,04	$[M+H]^{+1}$	99	2,16E+08	2
Kaempferol 3-O-glucuronide	Flavonoids glucoronides	C21 H18 O12	-0,81	462,07945	463,08673	9,821	$[M+H]^{+1}$	98,4	1,59E+06	2

Miquelianin (Quercetin 3-O-glucuronide)	Flavonoids glucoronides	C21 H18 O13	0,15	478,07481	477,06753	8,703	$[M-H]^{-1}$	96,8	1,51E+08	1
Quercetin-3-O-Arabinoside (Guajaverin)	Flavonoids glycosides	C20 H18 O11	-1,04	434,08446	435,09174	9,378	$[M+H]^{+1}$	99,2	2,36E+07	2
Kaempferol-3-O-galactoside (Trifolin)	Flavonoids glycosides	C21 H20 O11	-0,74	448,10023	449,10751	9,404	$[M+H]^{+1}$	99,2	1,16E+07	2
Myricetin 3-O-galactopyranoside-1	Flavonoids glycosides	C21 H20 O13	0,69	480,09072	479,08345	7,306	$[M-H]^{-1}$	95,6	1,20E+07	2
Myricetin 3-O-galactopyranoside-2	Flavonoids glycosides	C21 H20 O13	0,84	480,09079	479,08352	7,464	$[M-H]^{-1}$	95,1	6,69E+06	2
Quercetin-3-O-galactoside (Hyperoside)	Flavonoids glycosides	C21 H20 O12	0,33	464,09563	463,08834	8,762	$[M-H]^{-1}$	99,5	5,08E+08	2
Quercetin-3-O-glucoside (Isoquercitrin)	Flavonoids glycosides	C21 H20 O12	0,02	464,09549	463,08818	8,57	$[M-H]^{-1}$	99,5	9,25E+08	1
Quercetin-3-O-rhamnoside (Quercitrin)	Flavonoids glycosides	C21 H20 O11	-1,11	448,10007	449,10734	9,905	$[M+H]^{+1}$	98,7	1,42E+08	2
Quercetin-malonylglucoside-1	Flavonoids glycosides	C24 H22 O15	-0,77	550,09545	551,10272	9,424	$[M+H]^{+1}$	98,3	1,08E+07	2
Quercetin-malonylglucoside-2	Flavonoids glycosides	C24 H22 O15	-0,7	550,09548	551,10276	10,321	$[M+H]^{+1}$	96,4	6,00E+06	2
Rutin	Flavonoids glycosides	C27 H30 O16	-0,79	610,1529	611,16018	8,11	$[M+H]^{+1}$	98,2	3,42E+06	1
Taxifolin 3-O-rhamnoside (Astilbin)	Flavonoids glycosides	C21 H22 O11	0,13	450,11627	449,10897	9,053	$[M-H]^{-1}$	95,2	1,04E+09	2
Hyperforin	Naphtodianthrones	C35 H52 O4	0,74	536,38695	535,37968	23,173	$[M-H]^{-1}$	96,2	2,82E+10	1
Adenine	Nucleotides and Bases	C5 H5 N5	-0,89	135,05438	136,06165	0,836	$[M+H]^{+1}$	99,8	2,34E+07	2
Adenosine	Nucleotides and Bases	C10 H13 N5 O4	-0,94	267,0965	268,10378	1,057	$[M+H]^{+1}$	99,9	3,64E+07	2
Guanine	Nucleotides and Bases	C5 H5 N5 O	-0,85	151,04928	152,05656	0,841	$[M+H]^{+1}$	85,9	2,52E+06	2
Betaine	Others	C5 H11 N O2	-1,48	117,07881	118,08608	0,8	$[M+H]^{+1}$	99,4	5,92E+07	2
Choline	Others	C5 H13 N O	-1,78	103,09953	104,10681	0,765	$[M+H]^{+1}$	99,2	6,05E+08	2
Nicotinamide	Others	C6 H6 N2 O	-1,11	122,04788	123,05515	1,025	$[M+H]^{+1}$	97,7	5,38E+06	2
5-O-Coumaroylquinic acid	Phenolic acid & derivatives	C16 H18 O8	0,77	338,10043	337,09315	4,37	$[M-H]^{-1}$	98,9	2,80E+08	2
Caffeic acid	Phenolic acid & derivatives	C9 H8 O4	-1,03	180,04207	163,03878	3,288	$[M+H-H_2O]^{+1}$	98,5	2,95E+07	1
Chlorogenic acid	Phenolic acid & derivatives	C16 H18 O9	-0,93	354,09475	355,10203	3,289	$[M+H]^{+1}$	99,5	1,01E+08	1
Methyl protocatechuate	Phenolic acid & derivatives	C8 H8 O4	0,58	168,04236	167,03508	9,022	$[M-H]^{-1}$	89,5	1,05E+06	2
Quinic acid	Phenolic acid & derivatives	C7 H12 O6	-0,16	192,06336	191,05608	0,814	$[M-H]^{-1}$	99,8	1,70E+09	2
Hypericin	Prenylated Phloroglucinols	C30 H16 O8	0,69	504,08487	503,07759	25,132	$[M-H]^{-1}$	97,2	9,65E+09	1
Caryophyllene oxide	Sesquiterpenes	C15 H24 O	-0,99	220,1825	203,17921	18,117	$[M+H-H_2O]^{+1}$	96,6	6,54E+07	2
Hexose disaccharide (Trehalose)	Sugars & Sugar Alchools	C12 H22 O11	0,06	342,11623	341,10895	0,792	$[M-H]^{-1}$	97,9	4,04E+07	2
Iditol	Sugars & Sugar Alchools	C6 H14 O6	-0,03	182,07903	181,07181	0,78	$[M-H]^{-1}$	96,6	1,86E+07	2
Pentitol	Sugars & Sugar Alchools	C5 H12 O5	0,47	152,06854	151,06125	0,789	$[M-H]^{-1}$	95,7	3,78E+06	2
Cinnamtannin B1	Tannins	C45 H36 O18	-1,5	864,18887	865,19614	6,805	$[M+H]^{+1}$	98,5	2,28E+06	2

Table S3: *List of putative compounds identified on the C18 column.*

Name	Class	Formula	Δ Mass [ppm]	Calc. MW	m/z	RT [min]	Reference Ion	MzCloud match	Area Average	ID level
Trigonelline	Alkaloids	C7 H7 N O2	-1,01	137,04754	138,05482	10,078	$[M+H]^{+1}$	99,9	4,75E+08	2
Tryptamine	Alkaloids	C10 H12 N2	-1,05	160,09988	144,08063	2,475	$[M+H-NH_3]^{+1}$	98,3	6,20E+06	2
Proline	Amino acid & derivatives	C5 H9 N O2	-1,37	115,06317	116,07045	12,126	$[M+H]^{+1}$	99,8	8,28E+08	1
Valine	Amino acid & derivatives	C5 H11 N O2	-1,15	117,07884	118,08612	12,454	$[M+H]^{+1}$	98,7	2,64E+08	1
Threonine	Amino acid & derivatives	C4 H9 N O3	-0,92	119,05813	120,06541	16,227	$[M+H]^{+1}$	93,8	4,06E+06	1
Isoleucine	Amino acid & derivatives	C6 H13 N O2	-0,87	131,09451	132,10179	8,912	$[M+H]^{+1}$	99,8	1,53E+08	1
Leucine	Amino acid & derivatives	C6 H13 N O2	-0,67	131,09454	132,10182	7,717	$[M+H]^{+1}$	99,4	9,69E+07	1
Glutamic acid	Amino acid & derivatives	C5 H9 N O4	-0,57	147,05307	148,06035	16,856	$[M+H]^{+1}$	98,8	5,84E+06	1
Phenylalanine	Amino acid & derivatives	C9 H11 N O2	-1,03	165,07881	166,08608	7,411	$[M+H]^{+1}$	95,3	1,41E+08	2
Tyrosine	Amino acid & derivatives	C9 H11 N O3	0,89	181,07405	180,06678	14,362	$[M-H]^{-1}$	87,4	2,83E+07	2
Tryptophan	Amino acid & derivatives	C11 H12 N2 O2	0,85	204,09005	203,08277	7,842	$[M-H]^{-1}$	93,7	3,42E+07	1
Prolylleucine	Amino acid & derivatives	C11 H20 N2 O3	-0,89	228,14719	229,15447	15,48	$[M+H]^{+1}$	88,7	2,07E+07	2
Kynurenic acid	Amino acid & derivatives	C10 H7 N O3	-0,9	189,04242	190,0497	3,727	$[M+H]^{+1}$	99	2,08E+07	2
Y-Aminobutyric acid (GABA)	Amino acid & derivatives	C4 H9 N O2	-1,23	103,0632	104,07048	15,68	$[M+H]^{+1}$	89,4	6,00E+07	2
Methionine sulfoxide	Amino acid & derivatives	C5 H11 N O3 S	-0,63	165,04586	166,05314	16,625	$[M+H]^{+1}$	91,5	1,80E+06	2
Biapigenin (Amentoflavone)	Biflavonoids	C30 H18 O10	-1,56	538,08916	539,09643	0,962	$[M+H]^{+1}$	97,3	1,66E+09	2
Biapigenin isomer	Biflavonoids	C30 H18 O10	-1,32	538,08929	539,09657	1,093	$[M+H]^{+1}$	97,3	1,16E+09	2
Glyceric acid	Carboxylic acids	C3 H6 O4	1,25	106,02674	105,01946	8,574	$[M-H]^{-1}$	92,8	1,20E+07	2
Guanidinobutyric acid	Carboxylic acids	C5 H11 N3 O2	-0,7	145,08502	146,0923	13,156	$[M+H]^{+1}$	90,4	2,25E+07	2
Indole-acrylic acid	Carboxylic acids	C11 H9 N O2	-0,68	187,0632	205,09703	7,845	$[M+NH_4]^{+1}$	91,9	8,09E+07	2
Methylmalonic acid	Carboxylic acids	C4 H6 O4	0,89	118,02671	117,01944	1,683	$[M-H]^{-1}$	93,8	5,23E+07	2
Nicotinic acid	Carboxylic acids	C6 H5 N O2	-0,99	123,03191	124,03918	3,132	$[M+H]^{+1}$	98,2	2,94E+06	2
Gentisic acid	Carboxylic acids	C7 H6 O4	0,73	154,02672	153,01944	1,275	$[M-H]^{-1}$	95,4	7,22E+07	2
Gluconic acid	Carboxylic acids	C6 H12 O7	0,65	196,05843	195,05115	16,861	$[M-H]^{-1}$	88,4	2,31E+07	2
Apigenin	Flavonoids	C15 H10 O5	0,7	270,05301	269,04574	0,848	$[M-H]^{-1}$	93,6	8,32E+06	2
Keampferol	Flavonoids	C15 H10 O6	0,87	286,04799	285,04071	0,905	$[M-H]^{-1}$	97,5	4,92E+07	1
Luteolin	Flavonoids	C15 H10 O6	0,94	286,04801	285,04073	0,986	$[M-H]^{-1}$	95	3,52E+07	1
Epicatechin	Flavonoids	C15 H14 O6	0,73	290,07925	289,07194	1,307	$[M-H]^{-1}$	98,3	1,17E+09	1
Catechin	Flavonoids	C15 H14 O6	0,9	290,0793	289,07201	1,119	$[M-H]^{-1}$	98,2	2,48E+08	1
Quercetin	Flavonoids	C15 H10 O7	0,55	302,04282	301,03554	1,078	$[M-H]^{-1}$	99,3	1,05E+09	1
Taxifolin	Flavonoids	C15 H12 O7	-0,82	304,05805	305,06533	1,562	$[M+H]^{+1}$	98,5	1,65E+08	2
Myricetin	Flavonoids	C15 H10 O8	0,72	318,0378	317,03052	1,311	$[M-H]^{-1}$	90,5	7,45E+07	2

Kaempferol 3-O-glucuronide	Flavonoids glucoronides	C21 H18 O12	-0,18	462,07974	463,08702	5,647	[M+H]+1	90,5	6,66E+06	2
Miquelianin (Quercetin 3-O-glucuronide)	Flavonoids glucoronides	C21 H18 O13	0,42	478,07494	477,06767	4,945	[M-H]-1	96,9	1,32E+09	2
Quercetin-3O-glucoside (co-eluted)	Flavonoids glycosides	C21 H20 O12	0,17	464,09555	463,08828	2,335	[M-H]-1	99,7	6,79E+09	1
Quercetin-malonylglucoside	Flavonoids glycosides	C24 H22 O15	-0,94	550,09535	551,10263	3,977	[M+H]+1	92,8	8,14E+07	2
Quercetin-3-O-Arabinoside (Guajaverin)	Flavonoids glycosides	C20 H18 O11	-0,98	434,08448	435,09176	1,691	[M+H]+1	99,2	2,91E+08	2
Quercetin-3-O-rhamnoside (Quercitrin)	Flavonoids glycosides	C21 H20 O11	-1,29	448,09998	449,10726	1,618	[M+H]+1	98,9	1,06E+09	2
Kaempferol-3-O-galactoside (Trifolin)	Flavonoids glycosides	C21 H20 O11	0,54	448,1008	447,09353	1,813	[M-H]-1	85	2,99E+08	2
Taxifolin 3-O-rhamnoside (Astilbin)	Flavonoids glycosides	C21 H22 O11	0,05	450,11623	449,10896	1,573	[M-H]-1	95	2,87E+09	2
Myricetin 3-O-galactopyranoside-1	Flavonoids glycosides	C21 H20 O13	-0,64	480,09008	481,09736	3,58	[M+H]+1	98,1	1,12E+08	2
Myricetin 3-O-galactopyranoside-2	Flavonoids glycosides	C21 H20 O13	0,96	480,09085	479,08358	3,569	[M-H]-1	95,7	1,72E+08	2
Kaempferol 3-O-arabinofuranoside (Juglalin)	Flavonoids glycosides	C20 H18 O10	-0,32	418,08986	419,09714	1,419	[M+H]+1	87,4	2,85E+06	2
Hyperforin	Naphtodianthrones	C35 H52 O4	0,74	536,38695	535,37968	0,724	[M-H]-1	96,3	1,74E+10	1
Adenine	Nucleotides and Bases	C5 H5 N5	-0,93	135,05437	136,06165	2,6	[M+H]+1	99,8	1,90E+08	2
Guanine	Nucleotides and Bases	C5 H5 N5 O	-0,83	151,04928	152,05656	4,726	[M+H]+1	99,3	1,16E+07	2
Adenosine	Nucleotides and Bases	C10 H13 N5 O4	-0,93	267,0965	268,10378	2,957	[M+H]+1	96,8	2,61E+08	2
Cytosine	Nucleotides and Bases	C4 H5 N3 O	-1,18	111,04313	112,05041	3,642	[M+H]+1	95,4	2,64E+06	2
Deoxyadenosine	Nucleotides and Bases	C10 H13 N5 O3	-1,03	251,10158	252,10886	2,385	[M+H]+1	99,5	1,78E+07	2
Methyladenosine	Nucleotides and Bases	C11 H15 N5 O4	-0,54	281,11225	282,11953	1,821	[M+H]+1	97,9	8,86E+06	2
Guanosine	Nucleotides and Bases	C10 H13 N5 O5	1,15	283,09199	282,08472	7,226	[M-H]-1	90	1,63E+07	2
Choline	Others	C5 H13 N O	-2	103,09951	104,10678	3,975	[M+H]+1	99,8	3,45E+09	2
Betaine	Others	C5 H11 N O2	-1,21	117,07884	118,08611	8,683	[M+H]+1	99,6	2,49E+08	2
Nicotinamide	Others	C6 H6 N2 O	-0,86	122,04791	123,05518	1,404	[M+H]+1	99,8	2,14E+07	2
Nicotinyl alcohol	Others	C6 H7 N O	-1,23	109,05263	110,05991	1,295	[M+H]+1	96,7	2,99E+06	2
Hydroxy-pyridinemethanol	Others	C6 H7 N O2	-0,75	125,04758	126,05486	1,356	[M+H]+1	91,9	5,50E+06	2
Cuminaldehyde	Others	C10 H12 O	-0,69	148,08871	149,09599	2,095	[M+H]+1	86,7	3,71E+06	2
Caffeic acid	Phenolic acid & derivatives	C9 H8 O4	-1,24	180,04203	181,04931	5,165	[M+H]+1	94,2	1,12E+07	2
Quinic acid	Phenolic acid & derivatives	C7 H12 O6	0,19	192,06342	191,05615	15,989	[M-H]-1	99,7	7,33E+08	2
5-O-Coumaroylquinic acid	Phenolic acid & derivatives	C16 H18 O8	0,7	338,1004	337,09312	3,084	[M-H]-1	99,5	1,55E+09	2
4-O-Caffeoylquinic acid	Phenolic acid & derivatives	C16 H18 O9	-0,89	354,09477	355,10204	5,184	[M+H]+1	87,1	4,58E+07	2
Hydroxycinnamic acid 1	Phenolic acid & derivatives	C9 H8 O3	0,72	164,04746	163,04019	1,085	[M-H]-1	93,6	5,53E+06	2

Hydroxycinnamic acid 2	Phenolic acid & derivatives	C9 H8 O3	0,79	164,04747	163,0402	0,988	$[M\text{-}H]^{-1}$	92,9	6,75E+06	2
Gallic acid	Phenolic acid & derivatives	C7 H6 O5	0,54	170,02161	169,01434	1,671	$[M\text{-}H]^{-1}$	98,8	1,71E+07	2
Feruloylquinic acid	Phenolic acid & derivatives	C17 H20 O9	0,08	368,11076	367,10348	2,599	$[M\text{-}H]^{-1}$	96,6	9,58E+06	2
Hypericin	Prenylated Phloroglucinols	C30 H16 O8	0,69	504,08487	503,07759	0,667	$[M\text{-}H]^{-1}$	97,0	1,11E+09	1
Pentitol 2	Sugars & Sugar Alchools	C5 H12 O5	0,85	152,0686	151,06133	5,776	$[M\text{-}H]^{-1}$	98,7	1,84E+07	2
Hexitol 2	Sugars & Sugar Alchools	C6 H14 O6	0,87	182,0792	181,07192	10,135	$[M\text{-}H]^{-1}$	94,2	1,30E+08	2
Pentitol 1	Sugars & Sugar Alchools	C5 H12 O5	0,71	152,06858	151,0613	4,52	$[M\text{-}H]^{-1}$	93,5	2,15E+07	2
Hexitol 1	Sugars & Sugar Alchools	C6 H14 O6	0,86	182,07919	181,07192	9,63	$[M\text{-}H]^{-1}$	99,5	2,63E+07	2
Trehalose	Sugars & Sugar Alchools	C12 H22 O11	0,1	342,11625	341,10896	16,484	$[M\text{-}H]^{-1}$	97,9	5,98E+07	2
Cinnamtannin B1	Tannins	C45 H36 O18	-1,43	864,18893	865,1962	5,246	$[M+H]^{+1}$	98,7	4,41E+07	2

Table S4: *List of putative compounds identified on the amide column.*
